# Clinical diagnosis and treatment of immune checkpoint inhibitors‐related endocrine dysfunction

**DOI:** 10.1111/1759-7714.13347

**Published:** 2020-02-11

**Authors:** Lian Duan, Linjie Wang, Hanping Wang, Xiaoyan Si, Li Zhang, Xiaowei Liu, Yue Li, Xiaoxiao Guo, Jiaxin Zhou, Huijuan Zhu, Li Zhang

**Affiliations:** ^1^ Key Laboratory of Endocrinology of National Health Commission, Department of Endocrinology Peking Union Medical College Hospital, Chinese Academy of Medical Science and Peking Union Medical College Beijing China; ^2^ Department of Respiratory, Peking Union Medical College Hospital Chinese Academy of Medical Science and Peking Union Medical College Beijing China; ^3^ Department of Laboratory, Peking Union Medical College Hospital Chinese Academy of Medical Science and Peking Union Medical College Beijing China; ^4^ Department of Ophthalmology, Peking Union Medical College Hospital Chinese Academy of Medical Science and Peking Union Medical College Beijing China; ^5^ Department of Gastroenterology, Peking Union Medical College Hospital Chinese Academy of Medical Science and Peking Union Medical College; ^6^ Department of Cardiology, Peking Union Medical College Hospital Chinese Academy of Medical Science and Peking Union Medical College Beijing China; ^7^ Department of Rheumatology, Peking Union Medical College Hospital Chinese Academy of Medical Science and Peking Union Medical College Beijing China

**Keywords:** Endocrine dysfunction, immune checkpoint inhibitors, immune‐related adverse events

## Abstract

As a new class of antitumor drugs, immune checkpoint inhibitors (ICIs) have shown remarkable efficacy toward the treatment of various malignant tumors. By virtue of their targets and mechanisms of action, ICIs can cause autoimmune and inflammatory effects, termed as immune‐related adverse events (irAEs) and unlike the adverse reactions of traditional therapies, irAEs are occult and not fixed, with some serious adverse reactions forcing patients to stop treatment which might even affect their survival. Therefore, with the wide clinical application of ICIs, clinicians need to fully understand the possible adverse reactions of these drugs and devise reasonable treatment strategies to improve the survival rate and therapeutic effects of patients receiving ICIs. In this article, we review the incidence, clinical manifestations, diagnosis and treatment of immune‐related endocrine events that may occur with the administration of ICIs.

## Introduction

The occurrence of immune checkpoint inhibitor (ICI)‐related endocrine system adverse reaction is mostly delayed, with the median time being nine weeks after the start of medication (5–36 weeks). The most common adverse reactions are pituitary and thyroid dysfunction which may also involve the adrenal gland, pancreas, and parathyroid glands, manifesting with primary adrenal insufficiency, autoimmune diabetes, and hypoparathyroidism.^1^, ^2^, ^3^ The time of ICI‐induced hypophysitis is six to 14 weeks, and the time of thyroid dysfunction is usually four to seven weeks.^4^ The recovery of endocrine function often takes a long time and is life‐threatening if not treated in time, therefore requiring early identification and appropriate treatment.

## Hypophysitis

### Incidence

Previous studies have reported that the incidence of hypophysitis in patients treated with ipilimumab was 1.5% to 17%, while the rate of hypophysitis induced by nivolumab was only 0.6% to 1.5%. Combined drug therapy resulted in a higher incidence of hypophysitis in patients treated with ipilimumab, while with nivolumab and pembrolizumab, the incidence was 4% to 12.8%, and 9.1%, respectively.^4^, ^5^ It has been found that cytotoxic T‐lymphocyte‐associated antigen 4 (CTLA‐4) is expressed in pituitary gland. The combination of CTLA‐4 monoclonal antibody and antigen can activate classical complement cascade reaction, which leads to type II hypersensitivity reaction causing the occurrence of pituitary inflammation.^6^

### Clinical manifestations

ICI‐induced hypophysitis can cause total pituitary dysfunction or isolated anterior pituitary hormone deficiency, with or without pituitary enlargement and only a small number of patients have localized symptoms caused by pituitary enlargement. The most common clinical manifestations are headache and fatigue and other manifestations include memory loss, visual impairment, dizziness, anorexia, nausea, diarrhea, tachycardia, hypotension, decreased sexual function and amenorrhea.^7^ Although most patients may have multiple pituitary hormone deficiencies, secondary hypothyroidism, hypogonadotropic hypogonadism, and secondary adrenal insufficiency are more common, with incidences of 93%, 86%, and 75%, respectively. Growth hormone (GH) and prolactin (PRL) are less affected and diabetes insipidus is less common compared to other autoimmune hypophysitis.^8^, ^9^ It is worth noting that some patients with hypophysitis can develop adrenal crisis, which could be life‐threatening. This condition is typical of low blood pressure or shock, fever, anorexia, nausea, vomiting, disturbance of consciousness, coma and electrolyte imbalance (such as hyponatremia, hyperkalemia) and needs to be differentiated from severe sepsis.^10^

### Diagnosis

The evaluation of pituitary function is crucial as the clinical manifestations of patients with hypophysitis are not specific and similar to the symptoms caused by tumor progression, and should be differentiated from other causes such as infection and brain metastasis. When the patient has symptoms such as moderate fatigue or headache, nausea, vomiting or diarrhea, dizziness, orthostatic hypotension or hyponatremia and hemodynamic instability, hypophysitis needs to be considered and the patient should be promptly screened for biochemical indicators such as blood electrolytes and fasting blood glucose. Pituitary function should also be evaluated including early morning fasting adrenocorticotropic hormone (ACTH) and blood cortisol, thyroid‐stimulating hormone (TSH) and free thyroxine (FT_4_), follicle‐stimulating hormone (FSH), luteinizing hormone (LH) and estradiol (E_2_) or testosterone (T), PRL, GH and insulin‐like growth factor 1 (IGF‐1). If the patient has a thirst, polyuria, and polydipsia, it is also necessary to simultaneously check blood sodium, blood osmotic pressure, urine osmotic pressure and urine specific gravity.^10^, ^11^, ^12^ Pituitary enlargement and pituitary stalk thickening can occur before clinical symptoms, and therefore nuclear magnetic resonance imaging of the sellar region of patients suspected with clinical hypophysitis need to be recorded to understand the extent of pituitary enlargement, and whether there is optic nerve compression or not should be investigated in order to exclude brain metastases. Due to the high rate of ipilimumab‐induced hypophysitis, the assessment of pituitary function is important in patients receiving ipilimumab.^13^

### Treatment

If the patient has clinical hypovolemia, hypotension, hyponatremia, hyperkalemia or hypoglycemia, adrenal insufficiency should be considered. If blood cortisol is reduced and ACTH is not significantly elevated, it could be an indication of secondary adrenal insufficiency. Retrospective studies have found that super‐physiological glucocorticoid therapy does not improve clinical symptoms and shortens the recovery of pituitary function compared with replacement dose therapy.^14^, ^15^ At the same time, the use of larger doses of hormones increases the risk of infection and hyperglycemia. Therefore, it is recommended to use hydrocortisone (10–30 mg/day) for treatment, divided into several doses. In the event of an adrenal crisis, severe hyponatremia or severe headache, immunomodulation therapy should be discontinued and a large dose of glucocorticoids should be given immediately such as intravenous administration of hydrocortisone 100 mg once every eight hours.^10^, ^11^, ^12^ If the result of the thyroid function test indicates that FT_4_ is decreased and TSH is lowered or is within the inappropriate reference range, secondary hypothyroidism may be diagnosed and it will be necessary to give levothyroxine sodium (L‐T_4_) replacement therapy with a recommended starting dose of 0.8 μg/kg/day. Patients age >60 years or with cardiovascular disease need to start with a small dose (12.5–25 μg/day) and after four weeks, the patient should be reviewed and the drug dose titrated according to the FT_4_ level.^10^, ^11^, ^12^ Before administering L‐T_4_ replacement therapy, adrenal insufficiency should first be excluded. If both are present, glucocorticoid must be added first as the thyroid hormone promotes the removal of glucocorticoids and only supplements L‐T_4_, which will aggravate adrenal insufficiency, and even induce adrenal crisis.

If there are no treatment contraindications, male patients can be supplemented with testosterone. Because estrogen is associated with the risk of certain malignancies and venous thrombosis, premenopausal women should be treated with estrogen replacement therapy with caution and patients with malignant tumors should be banned from growth hormone therapy.^11^, ^12^

Patients who receive a reasonable replacement therapy usually do not need to stop immunomodulatory therapy and are required to temporarily discontinue immunotherapy only when the patient has a pituitary enlargement leading to occupying symptoms (such as visual impairment and headache) and adrenal crisis.^10^, ^12^ Retrospective studies have found that 63%–85% of patients with thyroid function can return to normal, approximately half of male patients with gonad function can recover, but that adrenal function is rarely restored. In patients with ICI‐induced hypophysitis, pituitary enlargement can be almost restored, with an average recovery time of around 15 weeks (2–27 weeks).^7^, ^8^

## Thyroid dysfunction

### Incidence

Unlike hypophysitis, ICI‐induced thyroid dysfunction is more common in anti‐programmed cell death‐1 (PD‐1) monoclonal antibody (anti‐PD‐1 mAb), with an average onset of six weeks after the first treatment. In a meta‐study, the rates of anti‐CTLA‐4, anti‐PD‐1, and anti‐programmed cell death‐ligand 1 (anti‐PD‐L1)‐induced hypothyroidism were 3.8%, 7.0%, and 3.9%, respectively, and the rate of hypothyroidism caused by nivolumab combined with ipilimumab was as high as 13.2%.^4^ The rates of anti‐CTLA‐4, anti‐PD‐1, and anti‐PD‐L1 induced hyperthyroidism were 1.7%, 3.2%, and 0.6%, respectively, and the rate of nivolumab combined with ipilimumab caused hyperthyroidism was 8.0%.^4^

The mechanism by which ICI induces thyroid dysfunction is not clear. Previous studies have found that CTLA‐4 gene polymorphisms are associated with autoimmune thyroid diseases, such as Graves' disease and Hashimoto's thyroid disease. Another clinical study has shown that ICI‐induced thyroid dysfunction may be associated with cytotoxic T lymphocyte‐mediated destruction of thyroid tissue.^16^ Although some researchers believe that most patients with ICI‐induced thyroid dysfunction have thyroid autoantibodies, there is no conclusive correlation between the two and this aspect needs further confirmation by prospective studies.^17^

### Clinical manifestations

Because of the routine detection of thyroid function, ICI‐induced abnormal thyroid function is generally diagnosed at an early stage. Patients usually have no symptoms or mild symptoms and most patients who visit a doctor do so because of symptoms of thyrotoxicosis, such as tachycardia, sweating, diarrhea and weight loss. Clinical manifestations of hypothyroidism include fatigue, constipation, chills, dry skin and weight gain. Although most patients have mild clinical manifestations, very few patients need to stop ICI treatment or initiate immunosuppressive therapy.^18^ However, there have been a few reports of ICI treatment‐induced thyroid crisis or severe hypothyroidism.^19^, ^20^

### Diagnosis

A diagnostic thyroid function test can help determine the patient's thyroid state. For example, if FT_4_ is decreased and TSH is elevated, primary hypothyroidism can be diagnosed, if FT_4_ is elevated and TSH is reduced, thyrotoxicosis is diagnosed, if FT_4_ is decreased and TSH is also decreased or is within the normal range, central hypothyroidism should be considered. The pituitary function should be further evaluated to determine if it is ICI‐induced hypophysitis. ICI‐induced abnormal thyroid function occurs in a short period of time after treatment. Some researchers recommend that thyroid function should be tested before each dose of ICI treatment and before the first five cycles of treatment, and thereafter after every three months of review.^12^, ^21^ Each time when the condition is being assessed attention should be paid to the symptoms associated with abnormal thyroid function. In addition, it should be noted that the use of iodine‐containing contrast agents for enhanced CT examination may affect the results of thyroid function test in the patient and it may no longer be possible to check the iodine absorption rate to identify thyroiditis (lower iodine absorption rate) or Graves' disease (increased iodine absorption rate) caused by thyrotoxicosis. If the patient has a thyroid‐related eye disease or goiter, the TSH receptor antibody should be screened. For patients who have not used iodine‐containing contrast agents recently, it is recommended that an iodine absorption test should be performed to assist diagnosis.^12^

### Treatment

Patients with abnormal thyroid function usually do not need to stop ICI treatment.^16^, ^18^ If it is primary hypothyroidism, L‐T_4_ needs to be supplemented and for specific treatment options, secondary hypothyroidism should be checked. If the patient presents with thyrotoxicosis (e.g., heart rate >100 bpm and no hypotension), beta‐blockers can be used shortly to improve symptoms. Since most patients with thyrotoxicosis will gradually progress to hypothyroidism (mean time is six weeks), it is recommended to review the thyroid function every two to three weeks. If the patient has hypothyroidism, beta‐blockers should be discontinued and L‐T_4_ should be supplemented to maintain normal condition. In elderly patients with cardiovascular disease and with a risk of severe thyrotoxicosis, short‐term usage of large doses of glucocorticoids (oral prednisone 1 mg/kg/day, one to two weeks) should be prescribed. If the TSH receptor antibody is positive or the thyroid iodine absorption is hyperactive, antithyroid drugs (such as methimazole) should be given.^12^, ^21^

## Primary adrenal insufficiency

### Incidence

Some studies reported that the incidence of autoimmune adrenalitis was only 0.7% (43/5831), and the incidence rate in combination therapy patients was 4.2%. Some researchers speculated that the combination of CTLA‐4 and PD‐1/PD‐L1 plays an important role in the occurrence of adrenal inflammation.^4^

### Clinical manifestations and treatment

Adrenal insufficiency should be considered when patients develop symptoms or signs such as fatigue, hypovolemia, hypotension, hyponatremia, hyperkalemia, hypoglycemia, fever, abdominal pain, skin pigmentation, and weight loss. Attention should be paid to the patient's vital signs and synchronous detection of early morning fasting cortisol and ACTH should be performed. When blood cortisol is decreased and ACTH is increased, primary adrenal insufficiency is diagnosed. If blood cortisol and ACTH are reduced, it is consistent with secondary adrenal insufficiency. If the patient has no obvious clinical manifestations, immunomodulatory therapy can be continued and hydrocortisone should be given at 10–30 mg/day, twice orally. If the patient has symptoms such as fatigue and anorexia but no hemodynamic instability immunomodulatory therapy needs to be stopped and hydrocortisone should be given at 10–30 mg/day, twice orally. If the patient develops adrenal crisis such as hypotension shock, dehydration, disturbance of consciousness, abdominal pain, vomiting and fever, immunomodulatory therapy should be stopped and hydrocortisone at 100 mg once every eight hours should be administered. If the diagnosis is of primary adrenal insufficiency, mineralocorticoid (hydrocortisone 0.05–0.2 mg/day) should be added. As adrenal insufficiency is difficult to recover, patients often need long‐term hormone replacement therapy, and patients and their families should be taught to increase the dose of glucocorticoids (usually two to three times the replacement dose) especially when associated with stress.^10^, ^11^, ^12^, ^21^

## Autoimmune diabetes mellitus

Gauci *et al*. retrospectively analyzed 24 cases of patients with ICI‐induced autoimmune diabetes, 50% (11/22) of which were positive for autoantibodies, and the average time for clinical manifestations was 8.5 weeks after the initial treatment (one week to 12 months). Further, 75% (18/24) of patients started with diabetic ketoacidosis (DKA) and all patients had elevated glycated hemoglobin, with 10 patients having significantly lower or no detectable C‐peptide levels, suggesting faster islet β‐cell failure.^22^ Once diagnosed, it is necessary to initiate insulin therapy immediately and give the basic combined meal insulin, and if necessary, also give a subcutaneous insulin pump to control blood glucose level. If the patient develops DKA, they should be given rehydration, continuous insulin infusion, and attention should be given to arterial blood gas, pH and electrolyte imbalance. Also, immunomodulation should be immediately stopped until blood glucose level is effectively controlled.^12^, ^21^, ^23^

## Hypoparathyroidism

ICI‐induced hypoparathyroidism was first reported in 2017. A 73‐year‐old male patient with metastatic melanoma developed severe hypocalcemia (blood calcium 5 mg/dL) after treatment with ipilimumab in combination with nivolumab. The patient had hyperphosphatemia (blood phosphorus 6.6 mg/dL), a significantly reduced level of hypoparathyroid hormone (<1 pg/mL), followed by thyrotoxicosis, and gradually developed primary hypothyroidism. After follow‐up to four months, the patient's thyroid and parathyroid function failed to recover, requiring L‐T_4_, calcium, and calcitriol replacement therapy.^24^

## Conclusions

The pathogenesis of ICI‐induced endocrine dysfunction and the predictors of adverse reactions have yet to be confirmed by more clinical studies. Clinicians and patients need to understand that most endocrine dysfunctions cannot be restored. Multidisciplinary collaborations are required to develop individualized treatments and programs and patients should receive long‐term follow‐up. Some endocrine emergencies such as adrenal crisis, if not identified early and treated appropriately, may endanger the patient's life. Therefore, proper attention should be given to the assessment of endocrine function and endocrinologists need to participate in the diagnosis and follow‐up of patients.

## Disclosure

The authors declare that they have no potential conflicts of interest, financial interests, relationships and affiliations relevant to the subject of their manuscript.
